# Parameter estimation of the structured illumination pattern based on principal component analysis (PCA): PCA-SIM

**DOI:** 10.1038/s41377-022-01043-9

**Published:** 2023-02-08

**Authors:** Xin Chen, Yiwei Hou, Peng Xi

**Affiliations:** 1 Department of Biomedical Engineering, College of Future Technology, Peking University, 100871 Beijing, China; 2 National Biomedical Imaging Center, Peking University, 100871 Beijing, China

**Keywords:** Biophotonics, Optical techniques

## Abstract

Principal component analysis (PCA), a common dimensionality reduction method, is introduced into SIM to identify the frequency vectors and pattern phases of the illumination pattern with precise subpixel accuracy, fast speed, and noise-robustness, which is promising for real-time and long-term live-cell imaging.

Among the optical super-resolution imaging techniques, structured illumination microscopy (SIM)^1–3^ technique is usually favored for long-term imaging of live cells with respect to its high spatiotemporal resolution, moderate labeling requirements, and low photon budget. Generally, high-quality and robust SIM reconstructed results rely heavily on post-processing algorithms^4,5^.

In the traditional SIM reconstruction method that commonly uses generalized Wiener filtering, it is crucial to solve and separate the frequency spectrums of the sample, then shift them to the correct position. To do this, it is essential to precisely know the parameters of the structured illumination pattern, which includes the illumination frequency vector, angle, initial phase, and modulation depth. Otherwise, substantial reconstruction artifacts would be introduced into the final reconstruction results^6,7^. Though the frequency vectors of the illumination pattern can be determined by using structured illumination generators with high precision and reproducibility such as spatial light modulators (SLM)^3,8,9^ and digital micromirror devices (DMD)^10,11^, it is hard to determine the initial phase through the hardware prior knowledge. Moreover, some factors such as sample movement and photobleaching would alter the pattern position in the raw images, confining the prior knowledge-based parameter estimation.

Thus, to exactly determine the illumination pattern’s parameters, many algorithm-based parameter estimation methods have been proposed during the post-processing, such as the commonly used cross-correlation (COR)^2,12^, phase of peak (POP)^13,14^, non-iterative auto-correlation (ACR)^15^, and image recombination transform (IRT)^16,17^. Only the COR-based estimation method can solve the frequency vector with subpixel precision. However, the iterative nature of COR inevitably leads to a longer computation time. Compared with COR, the other three non-iterative estimation methods have a faster speed for initial phase estimation but with lower precision, because they are based on the integer pixel frequency vector to estimate the phase. Besides, when the raw images with low signal-to-noise ratio (SNR) or weak modulation depth, none of the four algorithms could guarantee an acceptable precision of the phase estimation.

Now, writing in this issue of *eLight*, Jiaming Qian and colleagues at the Nanjing University of Science and Technology report for the first time an efficient and robust parameter estimation method based on principal component analysis (PCA)^18^. They demonstrated that PCA-SIM can achieve non-iteratively fast, accurate (below 0.01-pixel frequency vector and 0.1% of 2*π* relative phase under typical noise level), and robust parameter estimation at low SNR, which allows real-time super-resolution imaging of live cells in complicated experimental scenarios.

PCA is a widely used method of processing high-dimensional feature data, which utilizes singular value decomposition to decompose the matrix into a set of unrelated variables called principal components. In the work presented here, the authors introduced PCA as a “dimensionality reduction” tool for precise identification of noninteger pixel frequency vector and pattern phase while rejecting components that are uncorrelated to the desired, parameter-dominating “principal component.” Besides, to further accelerate PCA-SIM and enhance its noise robustness, they introduced an additional frequency-domain masking operator on the noisy 1-order spectrum before applying PCA (see Fig. 1). The advantage of PCA-SIM is that it can recover higher-quality super-resolution reconstruction results by instantly extracting the accurate illumination parameters from complex disturbances. In addition, they provided an open-source MATLAB code of their PCA-SIM algorithm and associated datasets, which will help users figure out how this method works.

**Fig. 1:**
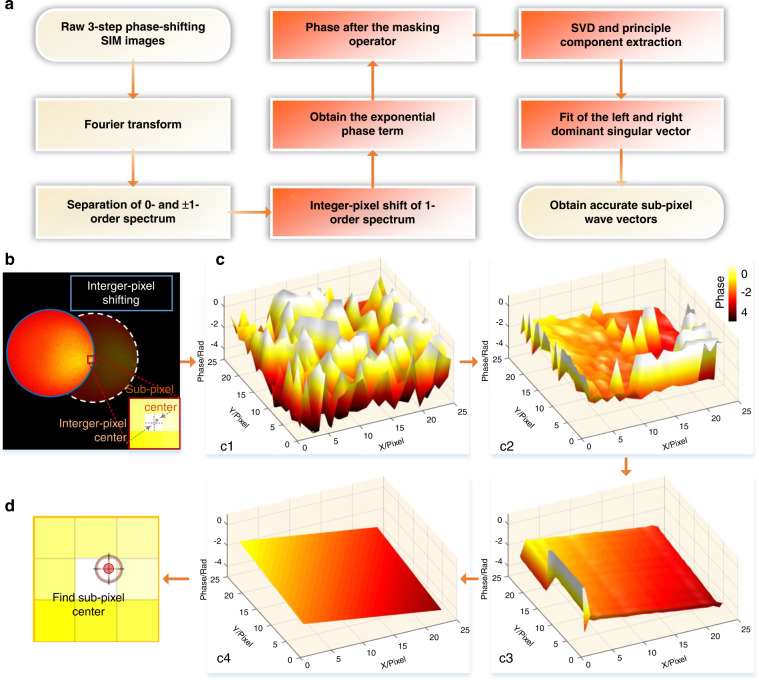
Schematic diagram of PCA-SIM. **a** Flow chart of the parameter estimation in PCA-SIM. **b**–**d** Output results of red boxes in **a**. **b** The 1^st^-order spectrum after being shifted interger-pixel frequency vector. **c** The phases of the phasor matrix obtained from **b** after different operations: the original phases (**c1**), phases after applying the masking operator (**c2**), phases after applying PCA (**c3**), and phases after least-squares fitting (**c4**). **d** Obtained frequency vector with sub-pixel accuracy

Jiaming Qian and co-authors demonstrated the superior super-resolution reconstruction capability and noise robustness of the PCA-SIM method by carrying out a set of comparative simulations and experiments. From the simulation results, it can be seen that PCA-SIM can obtain a more precise wave vector with an accuracy better than 0.01 pixel at low noise levels. When the noise level increases to 40 dBW, the errors of PCA-SIM can still be controlled within 0.2 pixels, which is lower than that (larger than 1 pixel) of COR. Besides, when processing the same raw data of BPAE cells, the time cost of PCA-SIM for parameter estimation is only 0.8119 ± 0.0011 s, while that of COR is 16.2810 ± 0.0355 s. Consistent with the simulation results, the experimental results showed that PCA-SIM outperforms SIM reconstruction algorithms that use COR, POP, ACR, or IRT for parameter estimation. Moreover, dynamic imaging scenes and results of a fixed BPAE cell sample with ambient light interference and artificial perturbations also demonstrated that PCA-SIM can provide immediate, high-quality super-resolution reconstruction in a complicated environment.

Unlike the video-rate immediate graphics processing unit-accelerated open-source reconstruction (VIGOR) method^19^, which calibrates the illumination parameters in advance, (e.g., by using the COR algorithm, and then reuses these parameters in the subsequent reconstruction). The successful realization of an instant parameter estimating strategy based on PCA demonstrates the feasibility of real-time SIM reconstruction, providing the potential to significantly improve the live-cell imaging performance of SIM under confined imaging conditions with external disturbances. It can be expected that the performance of the proposed scheme can be further promoted, such as the generalization to three-dimensional SIM, combination with regularization-based deconvolution techniques^20^, and so on, pushing its practicability to a higher level.
